# Dynamic MRI to quantify musculoskeletal motion: A systematic review of concurrent validity and reliability, and perspectives for evaluation of musculoskeletal disorders

**DOI:** 10.1371/journal.pone.0189587

**Published:** 2017-12-12

**Authors:** Bhushan Borotikar, Mathieu Lempereur, Mathieu Lelievre, Valérie Burdin, Douraied Ben Salem, Sylvain Brochard

**Affiliations:** 1 Laboratoire de Traitement de l’Information Médicale, INSERM U1101, Brest, France; 2 IMT Atlantique, Brest, France; 3 CHRU de Brest, Hôpital Morvan, Service de Médecine Physique et de Réadaptation, Brest, France; 4 Université de Bretagne Occidentale, Brest, France; 5 CHRU de Brest, Neuroradiologie, Imagerie Médico-Légale, Brest, France; Universite de Nantes, FRANCE

## Abstract

**Purpose:**

To report evidence for the concurrent validity and reliability of dynamic MRI techniques to evaluate *in vivo* joint and muscle mechanics, and to propose recommendations for their use in the assessment of normal and impaired musculoskeletal function.

**Materials and methods:**

The search was conducted on articles published in Web of science, PubMed, Scopus, Academic search Premier, and Cochrane Library between 1990 and August 2017. Studies that reported the concurrent validity and/or reliability of dynamic MRI techniques for *in vivo* evaluation of joint or muscle mechanics were included after assessment by two independent reviewers. Selected articles were assessed using an adapted quality assessment tool and a data extraction process. Results for concurrent validity and reliability were categorized as poor, moderate, or excellent.

**Results:**

Twenty articles fulfilled the inclusion criteria with a mean quality assessment score of 66% (±10.4%). Concurrent validity and/or reliability of eight dynamic MRI techniques were reported, with the knee being the most evaluated joint (seven studies). Moderate to excellent concurrent validity and reliability were reported for seven out of eight dynamic MRI techniques. Cine phase contrast and real-time MRI appeared to be the most valid and reliable techniques to evaluate joint motion, and spin tag for muscle motion.

**Conclusion:**

Dynamic MRI techniques are promising for the *in vivo* evaluation of musculoskeletal mechanics; however results should be evaluated with caution since validity and reliability have not been determined for all joints and muscles, nor for many pathological conditions.

## Introduction

The term ‘musculoskeletal disorder’ refers to conditions, diseases, and injuries of bones, joints and muscles. Musculoskeletal disorders can result from neurological diseases (e.g stroke, cerebral palsy) and orthopaedic disorders (e.g. anterior cruciate ligament injuries, osteoarthritis) that alter the human musculoskeletal system and impair its functions. The world-wide prevalence of musculoskeletal disorders is high, and they cause 21.3% of the total years lived with disability (ranked second after behavioral and mental health problems) [1–3]. Currently, standard static MRI sequences are used to provide a clinical diagnosis and an understanding of bone and tissue pathology. However, it could be hypothesized from a functional perspective, that abnormal or altered musculoskeletal mechanics cause musculoskeletal disorders. Furthermore, previous research has shown that images of static joint positions do not provide a comprehensive evaluation of the dynamic musculoskeletal system [4–9]. As a result, clinical, or even surgical treatments may be inappropriate. Understanding normal and impaired musculoskeletal function during motion is a high radiological, biomechanical and clinical priority. Accurate and reliable *in vivo* measurement of functional mechanics of the musculoskeletal system is thus necessary: 1) to understand normal joint mechanics in asymptomatic individuals, 2) to predict, detect or diagnose musculoskeletal disorders (e.g. scapholunate subluxation), and 3) to determine appropriate treatments for disorders using evidence based analysis.

Dynamic MRI techniques were originally developed for cardiovascular imaging to quantify blood flow and to study heart valve functions [10]. Dynamic MRI sequences for the quantification of functional joint motion were developed in the early 90’s [11–13]. As more dynamic sequences are being developed, they are becoming an integral part of image-based musculoskeletal modeling pipelines that rely heavily on dynamic imaging data to input joint kinematic parameters and predict patient specific outcomes [14]. However, controversial results have been reported for dynamic MRI based studies of joint mechanics in comparison with static studies. For example, the Achilles tendon moment arm determined using dynamic MRI by Sheehan FT [15] was much varied at larger ankle angles than reported previously by Manganais and colleagues [16] using static image based calculations. Despite an abundance of existing literature on dynamic MRI [14,17], no systematic reviews of the validity of these techniques have been carried out. Such a review is necessary to guide researchers and clinicians in the selection of the best available and validated techniques.

Concurrent validity and reliability provide valuable information for the interpretation of data. The aim of this systematic review was to report evidence of validity and reliability of dynamic MRI techniques to quantify *in vivo* joint and muscle mechanics. The global aim of this work was to identify gaps in the literature, to propose recommendations for the assessment of both healthy and impaired musculoskeletal function using current dynamic MRI techniques, and to make suggestions for future research in this field.

## Materials and methods

### Database search strategy

Articles published between 1990 and August 2017 were identified through a systematic search of the following five databases: (1) Web of science, (2) PubMed, (3) Scopus, (4) Academic search Premier, and (5) Cochrane Library. In order to ensure the search was systematic, the following combinations of keywords were used: 1) Keywords relative to acquisition method: “MRI”, “cine”, “dynamic”, “volumetric”, “velocity”, “in vivo” 2) “muscle”, “joint”, “bone” 3) “kinematics”, “displacement” 4) Keywords relative to metrological properties: “accuracy”, “reliability”, “repeatability”, “validity”. The guidelines by Sampson and McGowan [18] were used to reduce search errors. Search strings were formulated and tailored to the search syntax of each database to ensure a common search strategy (S1 Appendix). All keywords were truncated to check for variants in Pubmed, then the search was carried out without truncation. In this paper, validity refers to the general concept of concurrent validity [19] of the measurement error relating to joint kinematics or skeletal muscle motion properties between a reference method and the dynamic MRI method under evaluation. Reliability refers to intra/inter-rater/session reliability [20] of the dynamic MRI method used in the study.

### Study selection process

After removing duplicates from the search results, the titles and abstracts of the remaining studies were assessed by two reviewers independently to determine if they fulfilled the inclusion criteria. To be included in the review, studies had to fulfil three criteria: (1) the study was performed using a dynamic MRI imaging technique, (2) the study focused on joints or skeletal muscles and/or a moving phantom that mimicked joint or muscle movement, and (3) the study focused directly on quantifying concurrent validity and/or reliability. Exclusion criteria were: (1) the article was not published in English, (2) the article was categorized as a systematic or narrative review article or an editorial or a letter to the editor or as an abstract from conference proceedings, and (3) the article focused on moving or rotating phantoms but did not mimic skeletal joints or muscles. In the case of disagreement, consensus was reached by discussion.

To complete the review process, the references of the selected articles were also checked and articles found were included in the final selection. Four categories of data were extracted and presented in standardized tables: study population and joint/muscle studied, study description, dynamic tasks performed, dynamic MRI parameters, and results of concurrent validity and/or reliability.

### Quality assessment of selected studies

To the authors’ knowledge, no standardized tool for the assessment of quality of articles in this field currently exists. Thus, a customized quality assessment tool was developed based on three previously reported quality assessment tools for radiology and biomechanics related studies: 1) QUADAS—a tool for quality assessment of studies of diagnostic accuracy [21], 2) STROBE statement (STrengthening and Reporting of OBservational studies in Epidemiology) [22], and 3) quality assessment tools developed in recent systematic reviews of validity and reliability of joint motion analysis [23] and radiological assessment of hip geometry [24].

Two categories of quality were rated for each selected article (Table 1):1) intrinsic quality (Questions 1 to 11, Table 1), based on questions related to the study design, quality of reporting the methodology, and quality of reporting the results and findings/conclusion (maximum score 24); and 2) metrological evidence (Questions 12 to 17, Table 1), based on the questions related to quality of reporting the outcome measures and quality of metrological evidence to support the conclusions (maximum score 22). The total score (maximum 46) was converted into a percentage and named QAS (Quality assessment score). All the QAS values were rounded off to nearest integers for simplicity.

**Table 1:** Quality assessment score (QAS) questionnaire used to evaluate the quality of each selected article.

Sr. No.	Quality Question	Score Criteria
**1**	**Are the aims of the study clearly stated?**	**Clear (2) Partial (1) No (0)**
**2**	**Is there an adequate description of the patients/radiographs/ recruitment and controls?**	**Clear (2) Partial (1) No (0)**
**3**	**Was volunteer/patient consent obtained before the study?**	**stated (2)/ not stated (0)**
**4**	**Is the description of observer/reviewer/rater provided?**	**Clear (2) Partial (1) No (0)**
**5**	**Is there a clear description of equipment design and set-up?**	**Clear (2) Partial (1) No (0)**
**6**	**Is there a clear description of the measures?**	**Clear (2) Partial (1) No (0)**
**7**	**Is there a clear statement of statistical analysis or validity measures conducted?**	**Clear (2) Partial (1) No (0)**
**8**	**Are details about sample size calculation provided?**	**yes (2)/ partial (1)/ no (0)**
**9**	**Are the main outcomes of the study clearly stated?**	**Clear (2) Partial (1) No (0)**
**10**	**Are the key findings supported by the results?**	**Yes (2) Partial (1) No (0)**
**11**	**Is there a description of study limitations?**	**Clear (2) Partial (1) No (0)**
**12**	**Are the details of type of acquisition and acquisition parameters provided?**	**Clear (4) Partial (2) No (0)**
**13**	**Was the main aim metrological in terms of evaluation of validity and/or reliability?**	**both (4)/just one (2)/ no (0)**
**14**	**Was concurrent validity evaluated?**	**yes (4)/partial (2)/ no (0)**
**15**	**Was inter-observer reliability evaluated?**	**yes (4)/ without quantification/clinical relevance (2)/ no (0)**
**16**	**Was intra-observer reliability evaluated? OR Was intra-subject reliability evaluated?**	**yes (4)/ without quantification or clinical relevance (2)/ no (0)**
**17**	**Are the criteria for the avoidance of test-retest bias specified?**	**Yes with timing of tests or methodology specified (4)/ no (0)**

### Data analysis

Two observers independently reviewed the selected articles and rated the QAS. In case of significant disagreements in scores, consensus was reached by discussion. The QAS rated the overall quality of the selected article. To assess concurrent validity of techniques, the values of the results reported in the article were analyzed. Validity was considered excellent if errors were less than one millimeter or degree or cm/second, moderate if errors were in the order of one millimeter or degree or cm/second, and poor if errors were around, or greater than, two millimeters or degrees or cm/second. We acknowledge that this categorization has not been validated, however we used it to provide clarity when reporting the results. For the assessment of reliability, a Kappa coefficient (K), linear regression coefficient (r) or interclass correlation coefficient (ICC) between 0 and 0.60 was considered as poor, 0.60–0.80 as moderate, and 0.81–1.0 as excellent [25–28]. Due to the different statistical methods used in each article, it was impossible to directly compare or group the results. Thus, the results for validity and reliability were directly reported from the articles.

## Results

**Fig 1:**
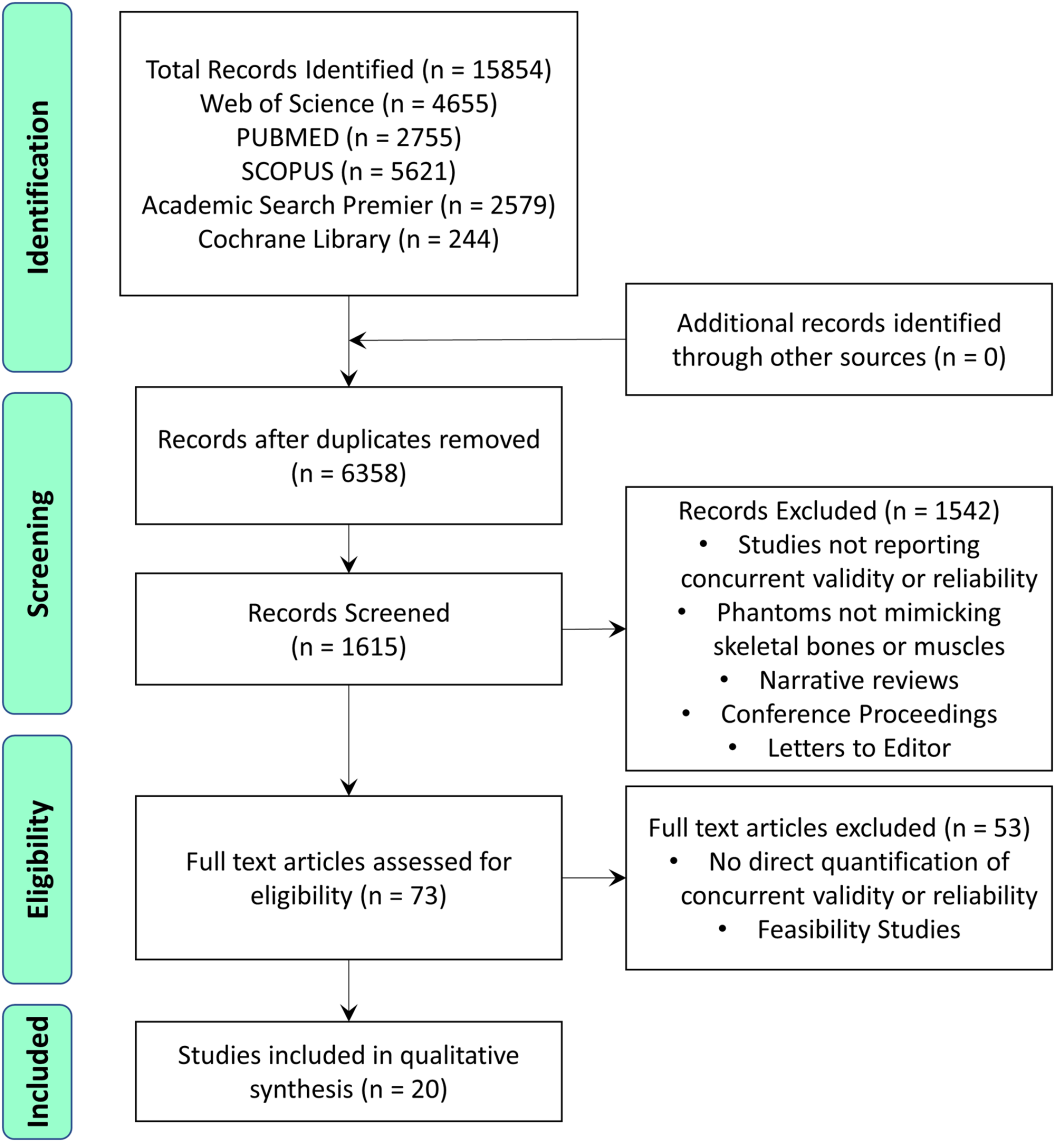
Flow chart of study selection.

The literature search identified 15854 articles from electronic databases, 6358 of which remained after removing duplicates. After screening titles and abstracts, 73 articles were found to be potentially eligible. Twenty articles were finally selected after verification of inclusion and exclusion criteria (Fig 1). The data were then summarized in four tables. Table 2 provides a description of study populations and designs, Table 3 provides details of tasks and measurement methods, Table 4 reports concurrent validity measures and Table 5 reports reliability measures. In the 20 studies, 1.5T and/or 3.0T MRI scanners were used, from the three major original equipment manufacturers (Philips, GE and Siemens), and for both open and closed bore types of scanner. This systematic review adheres to the PRISMA guidelines and a PRISMA checklist is available as a supplementary material (S2 Appendix)

**Table 2:** Description of study population and joint or muscle studied for each selected article.

Sr. No.	Study Name	Publication year	QAS (%)	Phantom used	Number of subjects	Mean age (years) ± SD	Gender (M = males, F = females)	Joint(s) or muscle(s) studied
**1**	Asakawa et al.[29]	2003	65	No	7 H	Adults	No data	Biceps brachii and triceps brachii muscles
**2**	Benham et al.[30]	2010	75	Moving Phantom	26 H	24.9 ± 5.1	13M/13F	Knee (patellofemoral and tibiofemoral joint)
**3**	Clark et al.[31]	2014	80	No	10 H	29 (range = 22 to 48)	5M/5F	Foot (ankle joint) phantom
**4**	Drace et al.[32]	1994	48	No	5 H	No data	No data	Forearm skeletal muscles
**5**	Drace et al.[33]	1994	48	Moving Phantom	4 H	No data	No data	Lower leg; forearm skeletal muscle; phantom
**6**	Draper et al.[34]	2008	85	Moving Phantom	6 H	26 ± 2	6F	Knee (patellofemoral joint)
**7**	Gilles et al.[35]	2005	53	No	6 H	No data	No data	Hip
**8**	Kaiser et al.[36]	2016	65	Moving Phantom	1H	18	F	Knee (tibiofemoral joint)
**9**	Langner et al.[37]	2015	68	No	14 H 38 NH	H = 28 ± 2.3 NH = 44 ± 11.2	4M/10F 15M/23F	Wrist (scapholunate)
**10**	Lin et al.[38]	2013	68	No	3 H	23 ± 0.0	No data	Knee (femur, tibia)
**11**	Moerman et al.[39]	2012	65	Moving Phantom	1H	No data	No data	Upper arm (biceps region)
**12**	Niitsu et al.[40]	1992	51	Moving Phantom	H (number not reported)	No data	No data	Leg skeletal muscles (various)
**13**	Pierrart et al.[41]	2014	61	No	4 H	34.2 (range = 30 to 45)	1M/3F	Shoulder (glenohumeral joint)
**14**	Powers et al.[42]	1998	73	No	12 H+3NH	range = 23 to 38	12 F	Knee (patelofemoral joint)
**15**	Rebmann et al.[43]	2003	66	No	8 H	33.0 ± 11.3	2M/6F	Knee (patello-femoral and tibio-femoral joints)
**16**	Sheehan et al.[44]	1998	53	Moving Phantom	5 H	No data	No data	Knee (patello-femoral joint)
**17**	Sheehan et al.[45]	2007	79	No	10 H	25.5 ± 3.9	9M/1F	Ankle (talocrural and subtalar joint)
**18**	Sinha et al[46]	2004	70	Moving Phantom	4 H + atrophied + rabbit	28 ± 8	3M/1F	Leg muscles (gastrocnemius, soleus)
**19**	Wang et al.[47]	2007	73	No	17 (7 H 10 NH)	No data	No data	Temporomandibular joint
**20**	Zhang et al.[48]	2011	71	No	30 H	24.5 ± 2.9	8M/22F	Temporomandibular joint

H: Healthy; NH: Non-healthy; QAS: Quality assessment score; SD: Standard Deviation.

**Table 3:** Dynamic tasks performed and magnetic resonance imaging (MRI) sequence parameters used for the selected articles.

Sr. No.	Study Name	Publication year	MRI Field Strength (Tesla)	MRI scanner name	Dynamic MRI technique used	Joint(s) or muscle(s) Studied	Motion Studied	Range of motion/amplitude	Plane of data acquisition	Metrological assessment	Reference method	MRI Sequence parameters	Triggering Mechanism	Scan time
**1**	Asakawa et al.[29]	2003	1.5	Signa CV/i MR scanner, GE^1^	Fast real-time PC	Biceps brachii and triceps brachii muscles	Elbow flexion	From full elbow extension to 45–90° of elbow flexion	Axial	Validity	Cine PC	**TR** = 30ms; **TE** = NR; **FOV** = 18cm; **Flip angle** = NR; **NEX** = NR; **Slice thickness** = 1cm; **Venc** = 10cm/s; **number of frames** = 112;	NR	10 sec.
**2**	Benham et al.[30]	2010	3.0	Achieva scanner, Philips^2^	Cine PC	Knee (patellofemoral and tibiofemoral joint)	**Validity:** LR; AP; Rot **Reliability:** LM; SI; AP; Flexion; Tilt; Varus	NR	Axial; Sagittal	Validity; reliability	Cine image	**TR** = 6.8ms; **TE** = 3.4ms; **FOV** = NR; **Flip angle** = 20°; **NEX** = 2; **Slice thickness** = 10mm; **Venc** = NR; **number of frames** = 3;	Optical trigger	2.06 and 1.08 min.
**3**	Clark et al.[31]	2014	3.0	Achieva scanner, Philips^2^	3D real-time, ultra-fast (turbo) gradient echo	Foot (ankle joint) phantom	Flexion, extension of the ankle	NR	Sagittal; Coronal	Validity; reliability	Trigonometry	**TR** = 2.731ms; **TE** = 1.34ms; **FOV** = 320*320mm; **Flip angle** = 10°; **NEX** = NR; **Slice thickness** = 4mm; **Venc** = NR; **number of frames** = 10–20;	NA	< 2 min.
**4**	Drace et al.[32]	1994	1.5	GE^1^ scanner	2D Gradient-echo cine PC MRI	Forearm skeletal muscles	Flexion, extension of fingers and wrist	NR	Axial; Longitudinal	Validity	Analytically derived trajectories	**TR** = 22-33ms; **TE** = 8-11ms; **FOV** = 16-24cm; **Flip angle** = 30°; **NEX** = 1–2; **Slice thickness** = NR; **Venc** = 5-30cm/s; **number of frames** = 16–32;	Plenthysmograph sensor	2~3 min.
**5**	Drace et al.[33]	1994	1.5	Signa imager, GE^1^	Cine PC	Lower leg; forearm skeletal muscle; phantom	**Phantom:** displacement in X,Y plane; **Subjects:** finger motion and wrist flexion and extension; ankle dorsi- and plantar flexion	NR	Axial	Validity	Analytically derived trajectories	**TR** = 22-33ms; **TE** = 8-15ms; **FOV** = 16cm; **Flip angle** = 30°; **NEX** = 1–2; **Slice thickness** = NR; **Venc** = 5-20cm/s; **number of frames** = 16–32;	Plenthysmograph sensor	NR
**6**	Draper et al.[34]	2008	0.5 and 1.5	1.5T Excite HD MRI scanner, GE^1^ and 0.5T Signa SP open-MRI scanner, GE^1^	Real-time MRI, single-slice spiral sequence	Knee (patellofemoral joint)	**Phantom:** trajectories of a phantom in X, Y plane; **Subjects:** patellar tilt and bissect offset of the knee	0° to 60°	Axial oblique	Validity; reliability	3D optical motion capture	**TR** = 21.4mm/28.5mm; **TE** = NR; **FOV** = 10cm/16cm; **Flip angle** = NR; **NEX** = NR; **Slice thickness** = 4.7mm; **Venc** = NR; **number of frames** = 47fr/s and 35fr/s;	NA	20 sec.
**7**	Gilles et al.[35]	2005	1.5	Intera MRI system, Philips^2^	bFFE sequence real-time MRI	Hip	Pelvis/femur relative trans. and rot.	NR	Coronal	Validity	3D sequential acquisition	**TR** = 3.5ms; **TE** = 1.1ms; **FOV** = 450*500mm; **Flip angle** = 80°; **NEX** = NR; **Slice thickness** = 10 mm; **Venc** = NR; **number of frames** = 6.7frame/sec;	NA	NR
**8**	Kaiser et al.[36]	2016	3.0	MR750, GE^1^	Dynamic SPGR-VIPR cine MRI	Knee (tibiofemoral joint)	flexion-extension of knee phantom	0° to 31.7°	Sagittal	Validity and reliability	Analytically derived trajectories	**TR** = 4ms; **TE** = 1.4ms; **FOV** = 24cm^3^; **Flip Angle** = 8°; **NEX** = NR; **Slice thickness** = 1.5mm; **Venc** = NR; **Number of frames** = 60;	Rotary encoder (MR310, Micronor, Newbury Park, CA)	5 min.
**9**	Langner et al.[37]	2015	3.0	Magnetom Verio, Siemens^3^	Cine MRI	Wrist (scapholunate)	Radial and ulnar abduction	From neutral position to extreme radial and ulnar abduction	Coronal	Validity; reliability	Arthroscopy; cineradiography	**TR** = 1.64ms; **TE** = 405.3ms; **FOV** = 196*196mm; **Flip angle** = NR; **NEX** = NR; **Slice thickness** = 10mm; **Venc** = NR; **number of frames** = NR;	Retrospective triggering using peripheral patient monitoring unit on the contralateral index finger	41 sec.
**10**	Lin et al.[38]	2013	3.0	Verio, Siemens^3^	Real-time MRI radial FLASH	Knee (femur, tibia)	Femur, tibia and knee trans. and rot. in X,Y,Z directions	0° to 80°	NR	Validity; reliability	3D static MRI	**TR** = 4.3ms; **TE** = 2.3ms; **FOV** = 192*192mm; **Flip angle** = 20°; **NEX** = NR; **Slice thickness** = 6mm; **Venc** = NR; **number of frames** = 103–119;	NA	NR
**11**	Moerman et al.[39]	2012	3.0	Intera scanner, Philips^2^	3D SPAMM tagged MRI	Upper arm (biceps region)	**Phantom:** Displacement in X,Y,Z directions; **Subjects:** biceps displacement in X,Y,Z directions	NR	Sagittal; Transversal; Coronal	Validity	Controlled indentor	**TR** = 2.39ms; **TE** = 1.16ms; **FOV** = 120*120*39mm; **Flip angle** =; **NEX** = NR; **Slice thickness** = NR; **Venc** = NR; **number of frames** = NR;	Scanner generated TTL pulse	177 ms
**12**	Niitsu et al.[40]	1992	1.5	Signa MR imager, GE^1^	Tagged MRI	Leg skeletal muscles (various)	**Phantom**: linear trans. or rot.; **subjects**: dorsi- and plantar flexion of the ankle	**Phantom**: 0 to 25mm and -30° to +40° (total 70°)	Sagittal; Coronal	Validity	Analytically derived trajectories	**TR** = 8.5–11.0ms; **TE** = 4.4–5.4ms; **FOV** = 128*256mm; **Flip angle** = 30°; **NEX** = 1; **Slice thickness** = 15mm; **Venc** = NR; **number of frames** = NR;	Triggered after audible burst of tagging pulses	19 to 24 sec.
**13**	Pierrart et al.[41]	2014	1.5	Signa system, GE^1^	Multi-slice 3D balanced gradient echo sequence real-time MRI	Shoulder (glenohumeral joint)	Arm abduction in the scapula blade direction	30° to 60°	Coronal oblique	Reliability	NR	**TR** = 3.6ms; **TE** = 1.3ms; **FOV** = 35*35cm; **Flip angle** = 65°; **NEX** = NR; **Slice thickness** = 10mm; **Venc** = NR; **number of frames** = 14;	NA	28 sec.
**14**	Powers et al.[42]	1998	1.5	64-MHz MR system, GE^1^	Kinematic MRI	Knee (patelofemoral joint)	Sulcus Angle, Tilt and Bisect Offset, rot. of the knee	0° to 45°	Axial	Reliability	NR	**TR** = 6.5ms; **TE** = 2.1ms; **FOV** = 38cm; **Flip angle** = 30°; **NEX** = 1; **Slice thickness** = 7mm; **Venc** = NR; **number of frames** = 6;	NA	45 sec.
**15**	Rebmann et al.[43]	2003	1.5	CX MR imager, GE^1^	CinePC1; cine PC2; fast-PC2	Knee (patello-femoral and tibio-femoral joints)	Rotations: tilt, flexion, twist	10° to 30°	Sagittal; Sagittal oblique	Reliability	NR	**TR** = NR; **TE** = minimum; **FOV** = 30*22.5 cm; **Flip angle** = 30°; **Slice thickness** = 10 mm; **Venc** = 20cm/sec; **number of frames** = 24; (1) **Cine-PC1: NEX** = 1; **TR** = 21 ms; (2) **Cine-PC2: NEX** = 2; **TR** = 21 ms; (3) **Fast-PC2: NEX** = 2; **TR** = 9 ms	retrospective triggering using optical trigger to detect motion	1) Cine-PC1: 2.49 min. 2) Cine-PC2: 5.33 min. 3) Fast-PC2: 2.48 min.
**16**	Sheehan et al.[44]	1998	1.5	Signa system, GE^1^	Cine PC	Knee (patello-femoral joint)	**Phantom**: X,Y trans. of the centroid of the fiducials; **Patients**: patellar flexion, twist and tilt w.r.t. femur	**Phantom**: NR; **Patients**: from full extension to 40° of flexion	**Phantom**: all planes **Patients:** Sagittal	Validity	Analytically derived trajectories	**TR** = 21ms; **TE** = min full; **FOV** = NR; **Flip angle** = 30°; **NEX** = NR; **Slice thickness** = NR; **Venc** = NR; **number of frames** = 24;	retrospective triggering using optical trigger to detect motion	4.12 to 8.19 min.
**17**	Sheehan et al.[45]	2007	1.5	LX-9.1M4 scanner, GE^1^	Fast cine PC MRI	Ankle (talocrural and subtalar joint)	Anatomic and X,Y,Z velocities of dorsi-plantarflexion of the foot relative to the tibia	From -13.5° to 37.2° (total 50.7°)	Sagittal oblique	Validity; reliability	Distance between vertices in the first time- frame	**TR** = 9.0ms; **TE** = 4.3ms; **FOV** = 30*30cm; **Flip angle** = 20°; **NEX** = 2; **Slice thickness** = 10.0mm; **Venc** = 30; **number of frames** = 1;	retrospective triggering using optical trigger to detect motion	3.42 min.
**18**	Sinha et al[46]	2004	1.5	Signa scanner, LX 8.7, GE^1^	PC MRI; Spin tag	Leg muscles (gastrocnemius, soleus)	Fluid velocity; lengthening and shortening of rabbit plantaris muscle; isometric contractions of the leg	Rabbit plantaris muscle: 6mm	Sagittal; Axial	Validity	Flowmeter; potentiometer	(1) cine PC: TR = 11.3ms; **TE** = 5.3ms; **FOV** = 22-32cm; **Flip angle** = 30°; **NEX** = 2; **Slice thickness** = 5-10mm; **Venc** = 10cm/s; **number of frames** = NR; (2) Spin tag: TR = 5.5ms; **TE** = 2.3ms; **FOV** = 32cm; **Flip angle** = 12°; **NEX** = 3; **Slice thickness** = 5mm; **Venc** = NR; **number of frames** = NR;	Retrospective gating	cine PC: 1.30 min. Spin tag: 2min.
**19**	Wang et al.[47]	2007	1.5	Avanto scanner, Siemens^3^	Dynamic HASTE sequence	Temporomandibular joint	Opening and closing of the mouth	Maximum opening and closing of the mouth	Sagittal	Reliability	NR	**TR** = 1180ms; **TE** = 65ms; **FOV** = 13cm; **Flip angle** =; **NEX** = NR; **Slice thickness** = 7mm; **Venc** = NR; **number of frames** = 30;	NA	35 sec.
**20**	Zhang et al.[48]	2011	1.5	Tim Trio scanner, Siemens^3^	Real-time radial FLASH gradient echo	Temporomandibular joint	Opening and closing of the mouth	Maximum opening and closing of the mouth	Sagittal oblique	Reliability	NR	**TR** = 4.3ms; **TE** = 2.2ms; **FOV** = 192*192mm; **Flip angle** = 20°; **NEX** = NR; **Slice thickness** = NR; **Venc** = NR; **number of frames** = 3fr/sec;	NA	28 sec.

2D: two-dimensional; 3D: three-dimensional; NR: Not reported; NA: Not Applicable; trans: Translations; rot: Rotations; ms: mili-seconds; sec: seconds; min: minutes; mm: millimeter; cm: centimeter; cm/s: centimeter per second; LR: Left-Right; AP: Anterior-posterior; LM: Lateral-medial; SI: Suerior-Inferior; Flex: Flexion; TR: Time to Recovery; TE: Time of Excitation; FOV: Field of View; NEX: Number of Excitations; Venc: Velocity Encoding; PC: Phase contrast; bFFE: balanced fast field echo; SPGR: spoiled gradient-recalled; VIPR: Vastly undersampled isotropic projection; HASTE: half-Fourier acquired single-shot turbo spin-echo; SPAMM: Spatial modulation of the magnetization; FLASH: fast low-angle shot; TTL: Transistor-Transistor Logic

^1^GE Medical Systems, Milwaukee, WI, USA

^2^Philips Medical Systems, Best, Netherlands

^3^Siemens Healthcare, Erlangen, Germany

**Table 4:** Results for concurrent validity.

Sr. No.	Study	Publication Year	Dynamic MRI Sequence used	Joint Studied	Method of Reference	Validity method	Statistical tool	Outcome variables	Validity results (Errors)			Range of motion
									X	Y	Z	
**1**	Asakawa et al.[29]	2003	Fast real-time PC	Biceps brachii and triceps brachii muscles	Cine PC	Mean error values	NR	Velocities in a region of interest within the biceps brachii	Mean error (from reported results) 1.47 cm/s			From full elbow extension to 45–90° of elbow flexion
**2**	Benham et al.[30]	2010	Cine PC	Knee (patellofemoral and tibiofemoral joint)	Cine images	Absolute difference	NR	LR and AP trans. and rot. of the phantom	Absolute error 0.16 mm	Absolute error 0.27 mm	0.46°	NR
**3**	Clark et al.[31]	2014	3D real-time, ultra-fast (turbo) gradient echo	Foot (ankle joint) phantom	Trigonometry	RMSE, Mean, SD, max absolute diff, CI	NR	Achilles tendon moment arms	Mean RMSE = 3.2 mm, mean = 2.9 mm, SD = 2.1 mm, max abs diff = 8.9 mm, 95% confidence = 2.3 to 3.5mm.			NR
**4**	Drace et al.[32]	1994	2D Gradient-echo cine PC MRI	Forearm skeletal muscles	Analytically derived trajectories	RMSE	NR	2D trans. of bovine muscle tissue placed on a phantom	RMSE 1 mm SD 0.2			NR
**5**	Drace et al.[33]	1994	cine PC	Lower leg; forearm skeletal muscle; phantom	Analytically derived trajectories	RMSE	NR	2D sinusoidal motion of a phantom	RMSE 0.04 mm.			NR
**6**	Draper et al.[34]	2008	Real-time MRI, single-slice spiral sequence	Knee (patellofemoral joint)	3D optical motion capture	RMSE	NR	Trajectories of a phantom in X,Y plane	1.5T: within 2mm for velocities slower than 217 mm/s; 0.5T: within 2 mm for velocities under 38 mm/s			0° to 60°
**7**	Gilles et al.[35]	2005	bFFE sequence real-time MRI	Hip	3D sequential acquisition	Mean error, SD	NR	Pelvis/femur relative trans. And rot.	Mean error = 1.8 mm and 1.3°; SD = 1 mm and 0.7°			NR
**8**	Kaiser et al.[36]	2016	Dynamic SPGR-VIPR cine MRI	Knee (tibiofemoral joint)	Tibio-femoral bone model	RMSE averaged over three trials	NR	Trans. and rot. of fiducial marker kinematics	RMSE 0.6 mm; 0.47°	RMSE 0.3 mm; 1.06°	RMSE 0.52 mm; 0.72°	0° to 31.7°
**9**	Langner et al.[37]	2015	Cine MRI	Wrist (scapholunate)	Arthroscopy and cineradiography	Sensitivity, specificity, and likelihood ratio	t-test; Fisher’s exact test; Bland-Altman plots	Scapholunate distance	Bland altman plot: good agreement; Sensitivity = 85%; Specificity = 90%; Positive and negative likelihood ratios: 8.5 and 0.16 respectively			From neutral position to the extreme radial and ulnar abduction
**10**	Lin et al.[38]	2013	Real-time MRI radial Flash	Knee (femur, tibia)	3D static MRI	Mean error, SD, RMSE	NR	Femur, tibia and knee trans. and rot. in X,Y,Z directions	Mean error: 0.3–0.9 mm and 0.1–0.2°; SD: 0.6–1.4 mm and 0.4–0.7°; RMSE: 0.7–1.7 mm and 0.4–0.7°	Mean error: 0.1–0.3 mm and 0.0–0.2°; SD: 0.4–0.8 mm and 1.0–1.4°; RMSE: 0.4–0.8mm and 1.0–1.4°	Mean error: 0.2–0.6 mm and 0.1–0.4°; SD: 0.4–0.6 mm and 1.1–1.8°; RMSE: 0.6–0.8 mm and 1.2–1.8°	0° to 80°
**11**	Moerman et al.[39]	2012	3D SPAMM tagged MRI	Upper arm (biceps region)	Controlled indentor	RMSE	Fitting of Gaussian models	Displacement of a phantom and skeletal muscle of the biceps in X,Y,Z directions	Phantom: displacement error = 0.44, SD = 0.59 mm; Volunteer: displacement error = 0.40, SD = 0.73 mm			NR
**12**	Niitsu et al.[40]	1992	Tagged MRI	Leg skeletal muscles (various)	Analytically derived trajectories	Linear correlation coefficient r; SD	NR	2D trans. and rot. of a phantom	r > 0.99; SD = 0.31 mm and 0.92°			Phantom: 0 to 25mm and -30° to +40° (total range 70°)
**13**	Sheehan et al.[44]	1998	Cine PC	Knee (patello-femoral joint)	Analytically derived trajectories	Average absolute error	NR	Phantom: X,Y trans. of the centroid of the fiducials	Mean 0.62 mm/0.55 mm	Mean 0.52 mm/0.36mm	NR	**Phantom**: NR; **Patients**: from full extension to 40° of flexion
**14**	Sheehan et al.[45]	2007	Fast cine PC MRI	Ankle (talocrural and subtalar joint)	Distance between vertices in the first time frame	Mean error	NR	Distance between the calcaneal, talar, and tibial vertices in each time frame relative to the absolute distance of vertices in the first time frame	Mean Calcaneus error: 0.0008mm, SD = 0.23 mm. Mean talus error: −0.0025mm, SD = 0.28 mm. Mean tibia error: 0.0006mm, SD = 0.21 mm			From -13.5° to 37.2° (total range 50.7°)
**15**	Sinha et al[46]	2004	PC MRI; Spin tag	Leg muscles (gastrocnemius, soleus)	Flowmeter; potentiometer	Coefficient of regression R	NR	Fluid velocity flow of the phantom and velocity of rabbit plantaris muscle	Phantom: R = 0.999; Rabbit: R = 0.94 in the sagittal scan and R = 0.98 in the axial scan			Rabbit plantaris muscle: 6mm

NR: Not reported; 2D: two-dimensional; 3D: three-dimensional; MRI: Magnetic resonance imaging; Trans: Translations; Rot: Rotations; mm: millimeter; cm/s: centimeter per second; mm/s: millimeter per second; PC: Phase contrast; FLASH: Fast low-angle shot; HASTE: half-Fourier acquired single-shot turbo spin-echo; SPAMM: Spatial modulation of the magnetization; SD: Standard deviation; RMSE: Root mean square error; r: correlation coefficient; R: Coefficient of regression; bFFE: balanced fast field echo; VIPR: Vastly undersampled isotropic projection; CI: Confidence intervals

**Table 5:** Results for reliability.

Sr. No.	Study	Publication Year	Dynamic MRI technique used	Joint Studied	Method	Number of Examiners	Examiner Qualifications and years of experience	Number of trials per session, number of sessions	Reliability coefficient	Outcomes variable	Reliability result		
											Inter-rater or inter-exam reliability	Intra-rater or intra-exam reliability	Range of motion/amplitude
**1**	Benham et al.[30]	2010	Cine PC	Knee (patellofemoral and tibiofemoral joint)	Subject repeatability	NA	NA	2 trials, 1 session	Grand mean of the standard deviation of the average kinematics	LM, IS, AP trans., flexion-extension, LM tilt, VV rot., and IE rot. of patellofemoral and tibiofemoral joint		Patellofemoral—< 0.73 mm and < 1.10°; tibiofemoral < 0.63 mm and < 0.78°	NR
**2**	Clark et al.[31]	2014	3D real-time, ultra-fast (turbo) gradient echo	Foot (ankle joint) phantom	Repeatability	NA	NA	14 trials, 1 session	Mean	Measurements of the moment arm for the validation apparatus	NR	Mean moment arm = 39.5 mm (SD = 3.5 mm)	NR
**3**	Draper et al.[34]	2008	Real-time MRI, single-slice spiral sequence	Knee (patellofemoral joint)	**Phantom:** repeatability; ***in vivo* study:** intraobserver and interobserver reliability	NA	NA	1 trial, 3 sessions	**Intraobserver reliability:** variance; **Interobserver reliability:** average RMS difference	**Intraobserver reliability:** measurement of bisect offset and patellar tilt; **Interobserver reliability:** two examiners measured kinematics from three extension cycles	RMS difference between 2 observer was 5.8% and 3.2°	**1.5T:** intraobserver repeatability was 1.7% and 0.37°; **0.5T:** intraobserver repeatability was 3.6% and 0.8°	0–60°
**4**	Kaiser et al.[36]	2016	Dynamic SPGR-VIPR cine MRI	Knee (tibiofemoral joint)	Tracking of fiducial markers on bones	NA	NA	3 trials, 1 session	precision	SD of differences	0.81° and 0.47 mm	NR	31.7° flexion
**5**	Langner et al.[37]	2015	Cine MRI	Wrist (scapholunate)	Interrater agreement	1 radiologist, 1 hand surgeon	7y for radiologist	2 trials, 1 session	Kappa	Scapholunate distance	Excellent interrater agreement for healthy and non healthy: K = 0.83 and 0.81 respectively	NR	From neutral position to the extreme radial and ulnar abduction
**6**	Lin et al.[38]	2013	Real-time MRI radial Flash	Knee (femur, tibia)	Repeatability	NA	NA	5 trials, 1 session	Average SD	Femur, tibia and knee trans. and rot. in X, Y, Z directions	NR	Trans. ranged from 0.2 mm to 1.2 mm and rot. ranged from 0.3° to 1.5°	0–80°
**7**	Moerman et al.[39]	2012	3D SPAMM tagged MRI	Upper arm (biceps region)	Random tag point location or tag displacement fields compared with the mean tag point locations or mean tag field displacement	NR	NR	NR	SD	Location and displacement of a phantom and skeletal muscle of the bicveps in X, Y, Z directions	NR	**Phantom**: location and displacement precision = 44 μm and 61 μm **Volunteer**: location and displacement precision = 92 μm and 91 μm	NR
**8**	Pierrart et al.[41]	2014	Multi-slice 3D balanced gradient echo sequence real-time MRI	Shoulder (glenohumeral joint)	Intraobserver reproducibility	NA	NA	6 trials, 1session	Difference between extreme and average value	X, Y, Z directions corresponding to the projection of humeral head center on glenoid coordinate system; SAS; GH abd.	NR	**Intra observer reproductibility:** X-2.5 mm, Y-2 mm, SAS = 1.4 mm, GH abd—1.2°	30–60°
**9**	Powers et al.[42]	1998	Kinematic MRI	Knee (patelofemoral joint)	Repeatability	1	NA	5 trials, 2 sessions	ICC (ICC (1) as per Baiko et al [49]	ICC of Sulcus Angle, Tilt and Bisect offset averaged on 5 measurements	NR	Sulcus Angle ICC = 0.67; Tilt ICC = 0.79; Bisect Offset ICC = 0.85	0–45°
**10**	Rebmann et al.[43]	2003	CinePC1; cine PC2; fast-PC2	Knee (patello-femoral-tibial)	SIEV, Precision	NA	NA	**SIEV:** 2 trials in 1 session; **Precision:** 10 analyses of post processed data	**SIEV:** absolute difference in patellofemoral and tibiofemoral orientation between 2 exams for the same subject; **Precision:** SD of the average orientation angles over 24 frames	**SIEV:** Tilt, flexion and twist for patellofemoral and tibiofemoral joints; **Precision:** Tilt, flexion and twist for femur and patella	**Fast PC SIEV:** from 1.6° to 2.4° for patellofemoral and from 0.8° to 2° for tibiofemoral; **cine PC1 SIEV:** from 2.3° to 4.7° for patellofemoral and from 1.3° to 3.5° for tibiofemoral; **cine PC2 SIEV:** from 2.4° to 6.1° for patellofemoral and from 1.6° to 2.8° for tibiofemoral	**Fast PC precision:** from 0.22° to 0.45° for femur and from 0.49° to 1.16° for tibia; **cine PC1 precision:** from 0.35° to 0.68° for femur and from 0.46° to 0.88° for tibia; **cine PC2 precision:** from 0.33° to 0.53° for femur and from 0.33° to 0.63° for tibia	10°–30°
**11**	Sheehan et al.[45]	2007	Fast cine PC MRI	Ankle (talocrural and subtalar joint)	Sequences repeated twice	NA	NA	2 trials/1 sessions	SD of the average	**Subject repeatability**: 3D kinematics of the talus and calcaneus relative to the tibia **Inter-Subject variability**: each kinematic variable	**Inter-subject variability:** ranged from 2.0 degrees to 5.9 degrees and 2.5 mm to 5.3 mm.	**Intra subject repeatability:** better than 1.8 degrees and 1.5 mm for the calcaneus relative to the tibia and 2.9 degrees and 1.2 mm for the talus relative to the tibia	From -13.5° to 37.2° (total range 50.7°)
**12**	Wang et al.[47]	2007	Dynamic HASTE sequence	TMJ	Interobserver reliability to compare reader confidence scores between examination types, GEE to evaluate differences between the examinations	NR	NR	NR	Kappa	Agreement between the dislocation rating of the TMJ for dynamic and static technique	K = 0.133 for dynamic examination and K = 0.231 for static examination	NR	Maximum opening and closing of the mouth
**13**	Zhang et al.[48]	2011	Real-time radial FLASH gradient echo	TMJ	Feasibility and interobserver variability	NR	NR	NR	Qualitative Score (1 good to 4 bad); Multi-rater kappa values	Relative positions of the mandibular condyle and articular disc	Good to almost perfect agreement and scores; artifact: K = 0.63; score: 1.01 ± 0.65; anatomical detectability: K = 0.89; score = 2.03 ± 0.71; disc displacement (K = 0.91) and condyle movement (K = 0.83).	NR	Maximum opening and closing of the mouth

NR: Not reported; NA: Not applicable; 2D: two-dimensional; 3D: three-dimensional; MRI: Magnetic resonance imaging; TMJ: Temporo-mandibular joint; SAS = width of subacromial space; GH abd = level of glenohumeral abduction; ICC: Interclass correlation coefficient; PC: Phase contrast; FLASH: Fast low-angle shot; HASTE: half-Fourier acquired single-shot turbo spin-echo; SPAMM: Spatial modulation of the magnetization; bFFE: balanced fast field echo; VIPR: Vastly undersampled isotropic projection;

### Quality assessment

The mean QAS of all the selected articles was 66% (± 10.46%) (Table 2). Two of the selected articles had a QAS of 80% or more and both these studies reported the concurrent validity of a real-time dynamic MRI technique [31,34]. Six studies had a QAS between 70% and 80% [30,42,45–48]. Seven studies had a QAS ranging from 60% to 70% [29,36–39,41,43]. Three studies had QASs between 50% and 60% [35,40,44]. The other two studies had QASs of 48% [32,33]. All the articles selected are presented to provide an all-inclusive review of the available literature on the metrological assessment of dynamic MRI techniques. Details of the scores of each article are provided in the supporting document S3 Appendix.

### Concurrent validity and reliability

Four studies [30,34,36,39] (mean QAS 73%) evaluated the concurrent validity of the technique in question using a moving phantom and later determined its reliability on healthy volunteers (Tables 4 and 5). Seven studies [29,32,35,40,44,46] (mean QAS 55%) evaluated only concurrent validity either using a moving phantom or another imaging technique as a gold standard (Table 4). Five studies [41–43,47,48] (mean QAS 69%) reported reliability using either repeated measures or multiple observers (Table 5). Four studies [31,37,38,45] (mean QAS 74%) reported both concurrent validity and reliability using measurements on healthy volunteers (Tables 4 and 5).

### Dynamic MRI techniques used and joints and muscles studied

Concurrent validity and/or reliability was determined for eight dynamic MRI techniques (Table 3): cine MRI [36,37], kinematic MRI [42], Ultrafast MRI [31], Cine Phase Contrast (PC) MRI [30,32,33,43–45], dynamic HASTE MRI [47], real-time MRI [34,35,38,41,48], real-time PC MRI [29], and Spin-tag or tagged MRI [39,40,46] (See S4 Appendix for a short description of each technique). The names of the sequences are reported as stated in the respective articles. The knee joint was the most frequently studied (seven studies), followed by the ankle and temporo-mandibular joints (two studies each), and the shoulder, wrist and hip joints (one study each). Three articles studied upper limb muscles and three studied lower limb muscles.

### Joint evaluations

#### Measurement of knee joint mechanics

Of the seven articles that studied the knee joint (Tables 3, 6 and 7), three reported concurrent validity and/or reliability using cine PC MRI [30,43,44] (mean QAS 65%), two using real-time MRI [34,38] (mean QAS 77%) and one each using kinematic MRI [42] (QAS 73%) and cine MRI [36] (QAS 66%).

**Table 6:** Concurrent validity for each joint and muscle studied.

Joint or skeletal muscle studied	Dynamic MRI techniques—Concurrent Validity							
	Cine	Kinematic	Ultrafast	Cine PC	dynamic HASTE	Real-Time	Real-time PC	Spin Tag or Tagged
**Knee**	trans +++ (1); rot +++ (1)			in-plane +++ (2); out of plane ++ (1)		trans and rot +++ (1); position trajectory ++ (1)		
**Ankle**			moment arm + (1)					
**Temporo-mandibular**								
**Shoulder**								
**Hip**						trans ++ (1); rotations ++ (1)		
**Wrist**								
**Lower limb muscles**				displacement +++ (1); displacement ++ (1)				muscle displacement +++ (2)
**Upper limb muscles**							velocity ++ (1)	muscle displacement +++ (1)

+++: Excellent evidence; ++: Moderate evidence; +: Poor evidence; Trans: Translations; Rot: Rotations; SLD: Scapholunate Dissociation; TMJ: Temporomandibular Joint; Numbers in brackets indicate the number of studies reporting the evidence.

**Table 7:** Reliability for each joint and muscle studied.

Joint or skeletal muscle studied	Dynamic MRI techniques—Reliability							
	Cine	Kinematic	Ultrafast	Cine PC	dynamic HASTE	Real-Time	Real-time PC	Spin Tag or Tagged
**Knee**	cartilage contact precision +++ (1)	bisect offset +++ (1); patellar tilt ++ (1)		trans +++ (2); rot ++ (2)		intra +++ (1); inter ++ (1)		
**Ankle**				intra ++ (1); inter + (1)				
**Temporo-mandibular**					TMJ open-close +++ (1)	inter (motion artifact) + (1); inter (disc motion) +++ (1)		
**Shoulder**						intra ++ (1)		
**Hip**								
**Wrist**	inter +++ (1); SLD +++ (1)							
**Lower limb muscles**				tracking ++ (1)				precision +++ (1)
**Upper limb muscles**								precision +++ (1)

+++: Excellent evidence; ++: Moderate evidence; +: Poor evidence; Trans: Translations; Rot: Rotations; SLD: Scapholunate Dissociation; TMJ: Temporomandibular Joint; Numbers in brackets indicate the number of studies reporting the evidence.

Among all the cine PC MRI techniques used, in-plane mean concurrent validity was excellent and out-of-plane mean concurrent validity was moderate to excellent [30,44] (mean QAS 64%) on 3.0T scanner. Furthermore, Benham et al. [30] reported that between no signal averaging and two signal averages, translational accuracy increases as much as 3.5 times, whereas rotational accuracy remains unchanged. Reliability of the cine PC MRI technique was reported by comparing knee kinematics (patellofemoral and tibiofemoral) from two acquisitions collected during same session. Reliability was moderate for rotations and excellent for translations [30,43] (mean QAS 71%).

For real-time MRI, the concurrent validity for tibio-femoral kinematics was moderate to excellent [38] (QAS 68%) [34] (QAS 85%) using a 3.0T [38] and 1.5T [34] scanner respectively. Intra-observer reliability was excellent and inter-observer reliability was poor for bisect offset and patellar tilt respectively [34] (QAS 85%).

For kinematic MRI, reliability was excellent for bisect offset measurements and moderate for patellar tilt and sulcus angle measurements [42] (QAS 73%). An average of two measurements within each session was recommended to produce adequate ICC values on bisect offset and patellar tilt whereas an average of four measurements was recommended to yield consistent sulcus angles.

For cine MRI, concurrent validity and reliability for tibiofemoral kinematic tracking were both excellent, using a 3.0T scanner [36]. The same study also reported excellent concurrent validity for determining tibiofemoral cartilage contact location.

#### Measurement of ankle joint mechanics

Ankle joint evaluations (Tables 3, 6 and 7) included talo-crural and subtalar kinematics [45] (QAS 79%) as well as quantification of muscle moment arms [31] (QAS 80%).

Sheehan and colleagues [45] (QAS 79%) reported moderate intra-subject reliability for the evaluation of ankle joint kinematics using Cine PC MRI on a 3.0T scanner. Clarke et al., [31] (QAS 80%) used ultrafast MRI to study the Achilles tendon moment arm using the ‘geometric method’ of measuring the distance from the joint axis to the muscle-tendon line-of-action and reported poor concurrent validity on a 3.0T scanner.

#### Measurement of temporo-mandibular joint (TMJ) mechanics

Since standard static clinical examinations cannot reliably assess TMJ disorders, dynamic MR imaging has become standard in the evaluation of TMJ problems. Two studies carried out metrological evaluation of dynamic MRI sequences based on quantitative parameters of TMJ mechanics (Tables 3 and 7). For dynamic HASTE sequence (half-Fourier acquired single-shot turbo spin-echo) acquired on a 1.5T scanner, Wang and colleagues [47] (QAS 73%) reported excellent reliability for the evaluation of maximal TMJ opening and closing. Zhang and colleagues [48] (QAS 71%) used real-time MRI with a radial data encoding scheme, and reported excellent reliability for visual assessment of the dynamic positions of the TMJ.

#### Measurement of shoulder, hip, and wrist joint mechanics

The metrological properties of dynamic MRI sequences at the shoulder, hip and wrist joints were each assessed in one study. For real-time MRI techniques, moderate reliability was reported for shoulder joint kinematics using a 1.5T scanner [41] (QAS 61%) and moderate concurrent validity was reported for hip translations and rotations using a 1.5T scanner [35] (QAS 53%). Gilles et al. further reported that an optimized protocol with reduced acquisition time and lowered image resolution (4 X 4 mm) resulted in poor concurrent validity for both translations and rotations of the hip joint [35].

For cine MRI, Langner et al. [37] (QAS 68%) reported excellent inter-rater reliability for the evaluation of scapholunate distance based on wrist joint motion and scapholunate dissociation (SLD) detection in healthy volunteers, as well as in individuals with clinically suspected SLD.

### Skeletal muscle mechanics

Six studies evaluated skeletal muscle motion using three different dynamic MRI techniques (Tables 2, 6 and 7). A spin tag or tagged MRI sequence was used in three studies [39,40,46] (mean QAS 62%), a cine PC MRI sequence was used in three studies [32,33,46] (mean QAS 55%), and a real-time PC MRI sequence was used in one study [29] (QAS 65%).

Using the Spin Tag technique, tagging pulse studies were performed for different lower leg muscles (gastrocnemius and soleus) [40] (QAS 51%) and for the biceps brachii [39] (QAS 65%) in healthy subjects. Both the studies showed excellent concurrent validity for the measurement of muscle displacement, as well as excellent reliability on a 1.5T scanner [40] and a 3.T scanner [39]. Sinha and colleagues [46] (QAS 70%) reported excellent concurrent validity for in-plane motion using MR-visible fluid following comparison of a velocity encoded PC MRI technique with spin tag MRI.

Drace and colleagues published two studies [32,33] (mean QAS 48%) of a velocity encoded cine PC MRI technique. In the first study [32] (QAS 48%), they reported excellent concurrent validity and excellent prediction of the sinusoidal displacements of a moving phantom, and in the second study [33], they reported moderate concurrent validity for 2D trajectory-tracking of skeletal muscles. Asakawa and associates [29] (QAS 65%) compared real-time PC MRI with cine PC MRI to determine the velocities of the biceps brachii, and found moderate concurrent validity for peak velocity values within the volunteers.

## Discussion

This systematic review reports current evidence regarding the metrological properties of dynamic MRI techniques for the measurement of joint and muscle mechanics. Eight dynamic MRI techniques identified from 20 selected articles were reported. Image acquisition techniques, output parameters, post-processing requirements, and metrological outcomes varied across studies. Moderate to excellent concurrent validity and reliability were reported for various MRI techniques in different studies for joints, moving phantoms, and muscles. However, only four out of 20 selected studies included subjects with musculoskeletal disorders, thus evidence for the metrological parameters of these techniques in clinical practice is currently lacking. Based on the current level of metrological evidence, the most valid and reliable techniques appear to be cine-PC and real-time MRI for joint mechanics and Spin tag MRI for muscle mechanics.

### Joint kinematics

The findings of this systematic review highlight that the concurrent validity of the different dynamic MRI techniques has not been evaluated for all joints (Tables 6 and 7). Concurrent validity was mostly evaluated using moving phantoms (Table 4), whereas reliability studies involved repeated measures in the same subject, or reporting observer reliability with image processing (Table 5). Overall, the largest number of joints were studied using cine PC and real-time MRI (three for cine PC and four for real-time), with good to excellent levels of validity. For knee joint kinematics, concurrent validity (2 studies, [30,44]) and reliability (2 studies [30,43]) were mostly evaluated using cine PC MRI compared to real-time MRI [38], cine MRI [36] and kinematic MRI [42]. However, excellent concurrent validity and reliability measures were reported for all the techniques used for knee joint evaluation. Fewer studies were carried out for the other joints. Furthermore, no studies evaluated concurrent validity for kinematic MRI or dynamic HASTE MRI, and no studies evaluated reliability for Ultrafast MRI and real-time PC MRI. Since the clinical evaluation of functional joint kinematics using dynamic MRI techniques is likely to expand (diagnosis, pre-operative planning, rehabilitation and clinical follow-up), it is necessary to assess the metrological evidence of the techniques used. Dynamic MRI techniques have been used to evaluate joint kinematics in the case of disorders of the knee joint [50–54], the wrist joint [37], the TMJ [47], the shoulder joint [55], and the spine [56–60]. However, no one dynamic MRI technique has been evaluated for concurrent validity and reliability for all joints. Further studies are thus required in both healthy subjects, and those with pathology.

### Skeletal muscle tracking

Many musculoskeletal and neurological disorders lead to changes in muscle properties and function that are still not well understood. Skeletal muscle tracking can be used to evaluate shear strain, tensile strain, and strain rate, along with regional deformations [32] and thus, could play a major role in understanding the pathophysiology of muscle disorders. However, very few studies and research groups use dynamic MRI techniques to study skeletal muscle disorders. For example, dynamic MRI techniques have been employed to determine impaired muscle mechanics in the Achilles tendon [61], gastrocnemius [62,63] and soleus muscles [63], however the validity of these techniques has been scarcely reported. Spin tag MRI is the only technique that consistently showed excellent concurrent validity and reliability for both upper and lower limb muscles. Tagged MRI sequences allow the measurement of deformation by tracking a tagged pattern on the muscles [39,46]. No other dynamic MRI techniques were used for muscle tracking/strain/displacement except cine PC MRI [46] and real-time PC MRI [29]. Furthermore, non-invasive measurement of the mechanical properties of muscles requires detailed *in vivo* measurements of skeletal muscles deformation. Thus, although the results of this study suggest spin-tag MRI is currently the most valid and reliable technique for the evaluation of muscle, further studies are required to confirm this.

### Limitations—Systematic review

This systematic review presents some limitations. The review protocol was not registered a priori in an international prospective register of systematic reviews, such as PROSPERO (https://www.crd.york.ac.uk/PROSPERO/). We did not use MeSH terms in the search strategy as MeSH terms were not consistent across the search engines and some search engines do not have controlled vocabulary (for e.g., Web of Science). However, the search strategy was cross-checked for common errors, according to the guidelines by Sampson et al. [18], and was made reproducible by providing the search strings used for each database (S1 Appendix). However, it is possible that certain keywords or word variants were missed. Certain databases, such as the Cochrane Library, automatically search for word variants in terms of linguistic variants, spelling (British vs American) variants, or even non-standard plural variants, however the other databases do not have this function, which could be a potential limitation of the search. Another limitation of this review was that the questionnaire (Table 1) used to determine QAS was not validated, although it was based on validated questionnaires. Thus, the QAS should be interpreted with caution.

### Limitations and improvements—Metrological studies

The main limitation of this review was the heterogeneity of MRI parameters, experimental designs, methods employed, and non-reported parameters due to manufacturer-specific sequences, which made it impossible to use a common scale for comparison. Even if studies used the same sequences, the parameters were heterogeneous since they are scanner dependent. Thus, although we recommend use of certain techniques, we cannot recommend a generalized set of parameters. To understand basic differences in these techniques, a brief methodological overview for each of these techniques with their trade names used by different manufacturers is provided in the S4 Appendix. Furthermore, not only did the metric quantification methods differ, different statistical methods were used to report concurrent validity (coefficient of regression (r), standard deviation, absolute differences, root mean square error, mean error values etc.) and reliability (standard deviation, absolute differences, interclass correlation coefficients, kappa statistics, root mean square, etc.).

Most *in vivo* tests were conducted on healthy volunteers. Only four studies (Table 3) included subjects with musculoskeletal disorders [37,42,46,47], and the data acquired was mostly used for feasibility or proof of concept. Despite the challenges relating to magnetism and scanner bore size constraints, it is now possible to mimic standing in an open MRI scanner or weight-bearing in a closed scanner. These conditions are considered to increase understanding of musculoskeletal disorders [17,64]. The literature suggests that researchers have succeeded in determining *in vivo* healthy joint kinematics for weight-bearing [65–67] and non-weight bearing conditions [15,68–71] that would evoke joint pain in pathological population. However, there are no studies of concurrent validity and reliability in persons with musculoskeletal disorders and abnormal joint kinematics. Future studies to evaluate dynamic MRI techniques should therefore involve patients with musculoskeletal disorders or mimic pathology.

With regard to the statistical analysis, which is a key point when reporting metrological studies, no exhaustive recommendations are available. However, for future reliability studies, we recommend reporting the standard error of measurement (SEM) or the minimal detectable difference of the measures. Reporting these metrics would allow the readers and users to attribute the observed difference to a true measurement of change, or a measurement error [27]. Furthermore, none of the studies carried out an a priori sample size calculation. This is important to ensure the study has adequate power [72,73].

This review highlighted that the most optimal way to evaluate the concurrent validity of dynamic MRI was by using motion phantoms that mimic joints or muscles. Search strategy found three studies [74–76] that reported the concurrent validity of cine PC MRI by using the known movement of specially designed motion phantoms, without mimicking joint or muscle motion. Since these studies did not fit in the aim of this systematic review, they were not included in the selected articles. We highly recommend the use of joint or muscle motion mimicking phantoms to evaluate all the dynamic MRI sequences using a single scanner in order to evaluate their concurrent validity.

### Future development

Future developments in this field can be classified into two categories: MRI sequence and post-processing techniques. Dynamic MRI sequences are evolving rapidly with advances in imaging technology. The typical fast imaging sequences based on balanced steady state free precession techniques, originally used for cardiac exams, are insufficient to obtain a total volume acquisition within a single breath hold for cardiac MRI [77]. A number of strategies have been developed to further reduce the acquisition time. These include, but are not limited to 1) k-t BLAST/SENSE (Sensitivity Encoding)/ASSET (Array coil Spatial Sensitivity Encoding) [78,79], 2) k-t FOCUSS [80], 3) parallel imaging techniques like GRAPPA (Generalised auto-calibrating partially parallel acquisition)/ARC (Autocalibrating Reconstruction for Cartesian imaging) [81], and 4) Echoplanar imaging (EPI). [78]. [82] All these imaging techniques and sequences are promising for the investigation of joint and muscle mechanics.

Although the focus of this review was not improving post-processing techniques, post-processing is key with regard to the feasibility and clinical utility of dynamic MRI. One such area that should be targeted is artifacts produced by eddy currents. In all types of imagery, eddy currents produce typical image artifacts that include image shearing, image scaling, and global position shifts. Thus, it is important to minimize the systematic error induced by eddy currents, which is possible using several techniques including 1) slotted coils and shields to interrupt current loops, 2) active shielding of gradients, and 3) image post-processing to correct for frequency/phase shifts. None of the selected articles reported the use of any of these techniques to minimize the eddy current error. However, one of the non-selected phantom studies [74] stated the use of post-processing techniques to reduce eddy current error.

### Perspectives for the evaluation of musculoskeletal disorders

Dynamic MRI-based evaluation of musculoskeletal disorders could have huge impact on understanding of the pathomechanics of the musculoskeletal system as well as to guide surgery [37] and rehabilitation [83]. Individuals with musculoskeletal disorders often experience joint pain and/or weakness during simple daily tasks or motions. Pain-inducing tasks would provide the most relevant dynamic MRI data, however, if such tasks are used, it is essential that the technique is quick and non-repetitive. While cine-PC and real-time MRI techniques stand out for the evaluation of skeletal joint mechanics, their use in the clinical setting is limited. For example, cine-PC MRI needs tasks to be repeated for up to two minutes (Tables 4 and 5) to acquire dynamic data. This is inappropriate in the case of pain. Real-time MRI can acquire dynamic data in single cycle, however requires slower joint motion, making the movement quasi-static. Future studies should focus on eliminating these limitations.

The most difficult challenge is to obtain physiological joint loading conditions inside the constrained space of the scanner, whether a horizontal close-bore system or upright open-bore system. Weight bearing MRI of joints is suggested to identify conditions that are otherwise challenging to diagnose using non-weight bearing MRI [64]. Weight bearing joint kinematics are different from non-weight bearing kinematics [4,5,7,9,84,85]. Furthermore, weight bearing joint kinematics are load dependent and change significantly with variations of the applied load [86]. Active *in vivo* joint kinematics are significantly different from passive or static analyses [8,87]. To reproduce physiological joint loading, special loading fixtures are needed which makes the experimental set-up complex and uncomfortable. Moreover, it is difficult to derive accurate and reliable joint kinematics from the acquired images because the quality of dynamic MR images is always lower than for static images. This is because fast image acquisition sequences with lower TR and TE values are typically used for dynamic MRI. Standardized processes for weight-bearing MRI have not yet been defined and their use for diagnosis, treatment and post-surgical follow-up remains to be specified.

In summary, dynamic MRI techniques may have potential to be used as clinical tools (for diagnosis or follow-up). However, there is a lack of metrological evidence for their use in the evaluation of musculoskeletal disorders. Moreover, due to the high costs involved, lack of standardization, lack of research demonstrating diagnostic value, post-processing time and complexity, manufactures are not developing and including standardized dynamic sequences for the study of musculoskeletal disorders. Thus, the role of dynamic MRI for the diagnosis of challenging cases is currently uncertain, and this technique is at an early stage of development. At the very best, dynamic MRI techniques can be used in the research setting to answer clinically important research questions such as understanding pain mechanisms [88] or evaluating functional anatomy [55,71] etc. Nevertheless, the results of this study regarding the validity and reliability of dynamic MRI techniques for the assessment of the musculoskeletal system are encouraging.

## Supporting information

S1 Appendix Search strings used for bibliographical search. (DOCX)

S2 Appendix PRISMA Checklist. (DOC)

S3 Appendix Table of QAS (quality assessment score). (XLSX)

S4 Appendix Brief review of dynamic MRI techniques. (DOCX)
