# Supplementary material for: Dynamic MRI to quantify musculoskeletal motion: A systematic review of concurrent validity and reliability, and perspectives for evaluation of musculoskeletal disorders
Source: PLoS One. 2017 Dec 12;12(12):e0189587. doi: 10.1371/journal.pone.0189587 (PMC5726646; doi:10.1371/journal.pone.0189587)
Supplement: S1 Appendix — (DOCX) [file pone.0189587.s001.docx]

**Pubmed – Medline Search String, 1 August 2017:**

(((“MRI”) AND (“cine” OR “dynamic” OR “volumetric” OR “velocity” OR “in vivo”) AND (“accuracy” OR “reliability” OR “repeatability” OR “validity”)) OR ((“MRI”) AND (“kinematics” OR “displacement”) AND (“accuracy” OR “reliability” OR “repeatability” OR “validity”)) OR ((“MRI”) AND (“muscle” OR “joint” OR “bone”) AND (“accuracy” OR “reliability” OR “repeatability” OR “validity”)) OR ((“MRI”) AND (“cine” OR “dynamic” OR “volumetric” OR “velocity” OR “in vivo”) AND (“kinematics” OR “displacement”) AND (“muscle” OR “joint” OR “bone”) AND (“accuracy” OR “reliability” OR “repeatability” OR “validity”))) NOT “contrast”

**Filters:** Title/Abstract; Humans; English; Date range 01/01/1990 – 01/08/2017

**Or a direct search string would be:**

(("MRI"[All Fields] AND ("cine"[All Fields] OR "dynamic"[All Fields] OR "volumetric"[All Fields] OR "velocity"[All Fields] OR "in vivo"[All Fields]) AND ("accuracy"[All Fields] OR "reliability"[All Fields] OR "repeatability"[All Fields] OR "validity"[All Fields])) OR ("MRI"[All Fields] AND ("kinematics"[All Fields] OR "displacement"[All Fields]) AND ("accuracy"[All Fields] OR "reliability"[All Fields] OR "repeatability"[All Fields] OR "validity"[All Fields])) OR ("MRI"[All Fields] AND ("muscle"[All Fields] OR "joint"[All Fields] OR "bone"[All Fields]) AND ("accuracy"[All Fields] OR "reliability"[All Fields] OR "repeatability"[All Fields] OR "validity"[All Fields])) OR ("MRI"[All Fields] AND ("cine"[All Fields] OR "dynamic"[All Fields] OR "volumetric"[All Fields] OR "velocity"[All Fields] OR "in vivo"[All Fields]) AND ("kinematics"[All Fields] OR "displacement"[All Fields]) AND ("muscle"[All Fields] OR "joint"[All Fields] OR "bone"[All Fields]) AND ("accuracy"[All Fields] OR "reliability"[All Fields] OR "repeatability"[All Fields] OR "validity"[All Fields]))) NOT "contrast"[All Fields] AND (hasabstract[text] AND ("1990/01/01"[PDAT] : "2017/08/01"[PDAT]) AND "humans"[MeSH Terms] AND English[lang])

**Cochrane Library search string, 1 August 2017**

((("MRI") and ("cine" or "dynamic" or "volumetric" or "velocity" or "in vivo") and ("accuracy" or "reliability" or "repeatability" or "validity")) or (("MRI") and ("kinematics" or "displacement") and ("accuracy" or "reliability" or "repeatability" or "validity")) or (("MRI") and ("muscle" or "joint" or "bone") and ("accuracy" or "reliability" or "repeatability" or "validity")) or (("MRI") and ("cine" or "dynamic" or "volumetric" or "velocity" or "in vivo") and ("kinematics" or "displacement") and ("muscle" or "joint" or "bone") and ("accuracy" or "reliability" or "repeatability" or "validity"))) not "contrast" in Trials and Methods Studies

**Web of Science Search string, 1 August 2017**

(TS = ((((“MRI”) AND (“cine” OR “dynamic” OR “volumetric” OR “velocity” OR “in vivo”) AND (“accuracy” OR “reliability” OR “repeatability” OR “validity”)) OR ((“MRI”) AND (“kinematics” OR “displacement”) AND (“accuracy” OR “reliability” OR “repeatability” OR “validity”)) OR ((“MRI”) AND ("muscle" OR "joint" OR “bone”) AND (“accuracy” OR “reliability” OR “repeatability” OR “validity”)) OR ((“MRI”) AND (“cine” OR “dynamic” OR “volumetric” OR “velocity” OR “in vivo”) AND (“kinematics” OR “displacement”) AND ("muscle" OR "joint" OR “bone”) AND (“accuracy” OR “reliability” OR “repeatability” OR “validity”))) NOT "contrast" )) *AND***LANGUAGE:** (English) *AND* **DOCUMENT TYPES:** (Article)

*Indexes=SCI-EXPANDED, ESCI Timespan=1990-2017*

**Scopus search string, 1 August 2017**

( TITLE-ABS-KEY ( ( ( "MRI" ) AND ( "cine" OR "dynamic" OR "volumetric" OR "velocity" OR "in vivo" ) AND ( "accuracy" OR "reliability" OR "repeatability" OR "validity" ) ) ) OR TITLE-ABS-KEY ( ( ( "MRI" ) AND ( "kinematics" OR "displacement" ) AND ( "accuracy" OR "reliability" OR "repeatability" OR "validity" ) ) ) OR TITLE-ABS-KEY ( ( ( "MRI" ) AND ( "muscle" OR "joint" OR "bone" ) AND ( "accuracy" OR "reliability" OR "repeatability" OR "validity" ) ) ) OR TITLE-ABS-KEY ( ( ( "MRI" ) AND ( "cine" OR "dynamic" OR "volumetric" OR "velocity" OR "in vivo" ) AND ( "kinematics" OR "displacement" ) AND ( "muscle" OR "joint" OR "bone" ) AND ( "accuracy" OR "reliability" OR "repeatability" OR "validity" ) ) ) AND NOT TITLE-ABS-KEY ( "contrast" ) ) AND DOCTYPE ( ar ) AND PUBYEAR > 1989 AND ( LIMIT-TO ( LANGUAGE , "English" ) )

**Academic Search Premier (EBSCO Host) search string, 1 August 2017**

AB (((“MRI”) AND (“cine” OR “dynamic” OR “volumetric” OR “velocity” OR “in vivo”) AND (“accuracy” OR “reliability” OR “repeatability” OR “validity”)) OR ((“MRI”) AND (“kinematics” OR “displacement”) AND (“accuracy” OR “reliability” OR “repeatability” OR “validity”)) OR ((“MRI”) AND ("muscle" OR "joint" OR “bone”) AND (“accuracy” OR “reliability” OR “repeatability” OR “validity”)) OR ((“MRI”) AND (“cine” OR “dynamic” OR “volumetric” OR “velocity” OR “in vivo”) AND (“kinematics” OR “displacement”) AND ("muscle" OR "joint" OR “bone”) AND (“accuracy” OR “reliability” OR “repeatability” OR “validity”))) NOT "contrast"

**Limiters** - Full Text; Date Published: 19900101-20171231; Language: English

**Narrow by Language:**- english

**Search modes** - Boolean/Phrase
