# Supplementary material for: Dynamic MRI to quantify musculoskeletal motion: A systematic review of concurrent validity and reliability, and perspectives for evaluation of musculoskeletal disorders
Source: PLoS One. 2017 Dec 12;12(12):e0189587. doi: 10.1371/journal.pone.0189587 (PMC5726646; doi:10.1371/journal.pone.0189587)
Supplement: S4 Appendix — (DOCX) [file pone.0189587.s004.docx]

**S3 APPENDIX 3**

This supplementary material provides information regarding the eight dynamic MRI sequences described in this review. The information about these sequences was collected from the selected articles as well as from other articles cited within the descriptions.

To produce an MR signal, a radiofrequency (RF) pulse is required to create a transverse magnetization vector which in turn induces current in the receiver coil. The most important parameters regarding the MRI signal generation are T1 (recovery time), T2 (decay time), T2* (decay time), TR (repetition time), and TE (time to echo). Briefly, T1 recovery is the time constant that determines the rate of longitudinal relaxation (direction of magnetic field), T2 decay is the time constant that determines the rate at which the excited protons lose phase coherence, TR (repetition time) is the amount of time between successive RF pulses applied, and TE (time to echo) is the time between the delivery of RF pulse and the receipt of echo signal (1,2). There is also a T2* decay time which is present due to the heterogeneity of the magnetic field at different places in the tissue (1). This is due to the presence of air, metallic objects, dental implants, calcium etc (1). As different tissues have different T1, T2, and T2* values, a radiologist can determine the tissue contrast for the clinical question by adjusting the TE and TR parameters to emphasize a particular type of contrast (1). For example, fat has a shorter T1 (recovers faster) and T2 (decays faster) than water and T2* decay occurs very quickly both in fat and water (1). Gradients are linear variations of the magnetic field strength in a selected region (3) and thus, three types of gradients are applied according to the imaging axis . Based on the orientation of the tissue, these become section-selective, phase-encoding, and frequency-encoding gradients (1). Thus, an MRI pulse sequence is a time description of RF pulse, gradients and data acquisition (4).

The time management of TR and TE using the radio frequency (RF) pulse and gradient waveforms and controlling the image acquisition at specific intervals within this waveform defines each of the pulse sequences. There are only two fundamental types of MR pulse sequences: Spin Echo and Gradient Echo (1,4,5). All the other MR sequences are variations of these sequences. Furthermore, MR pulse sequences can be either two dimensional (2D), with one section (slice) of a tissue acquired at a time, or three dimensional (3D), with a volume of multiple sections acquired in a single acquisition (1,4). Thus, the post-processing of the dynamic data is also dependent on whether the acquisition is 2D or 3D. Since most of the diagnostic and dynamic cardiac MR sequences were developed for imaging tissues infused with a contrast agent, typically a T1--weighted pulse sequence is used, and in most of the cases a gradient echo sequence is preferred over spin echo because of its shorter acquisition time. In a T1-weighted image sequence, the T1 contrast is accentuated and thus it is useful to reveal anatomical structures (6). In a T2- weighted pulse sequence, the T2 contrast is accentuated. These sequences are useful to investigate diseases, because most pathological tissues have a higher-than-normalwater and thus appear bright on T2-weighted images (6,7). Fast image acquisition using a gradient echo sequence is made possible by using very low TR and TE values (in the range of a few milliseconds up to a few hundred milliseconds). However, fast imaging techniques require thicker slices to produce enough signal for sufficient delineation of the tissue contrast, and also a lower number of slices to reduce the scan time. This leads to low resolution images and if the joint motion is not synchronized with the image acquisition times, then the resultant image is susceptible to motion artefacts. Furthermore, data acquired from dynamic sequences has systematic bias induced by eddy currents in terms of unwanted time-varying gradients and shifts in the main magnetic field (8–11).

**A.1 Cine MRI**

Cine MRI collects data continuously over several cycles and retrospectively sorts imaging data with a synchronization trigger in order to compensate for periodic motion. It is a well- established technique for cardiac imaging (12), imaging of the temporo-mandibular joint (13) as well as the wrist (14) and elbow joints (15). It allows dynamic acquisition with high spatial and temporal resolution. The sequence uses T2- weighted gradient echo pulse sequences with coherent transverse magnetization and pre-excitation refocused. The trade names on which cine MRI is based are PSIF (reversed fast imaging with steady-state precession) (Siemens), T2-FFE (T2 weighted fast field echo) (Philips) and SSFP (steady-state free precession) (GE).

**A.2 Cine-PC MRI**

Cine-PC MRI is a combination of Cine MRI and PC MRI. PC MRI is based on the detection of phase changes related to the movement of protons in a gradient magnetic field. By combining these two techniques, cine-PC MR imaging provides an anatomic image and three orthogonal velocity images (Vx, Vy, Vz) for each frame. PC imaging typically uses velocity induced phase shifts and thus velocity samples at a set of fixed points in space can be extracted. This velocity extraction over time creates a velocity map, which can be further used either for muscle trajectory computation (16) or for bone displacement quantification (17,18). The spatio-temporal velocity mapping needs tracking algorithms to implicitly or explicitly model the temporal and spatial variation of the velocity field to determine muscle trajectory. However, an extra post-processing step is needed to convert velocity mapping into spatial displacements to quantify bone kinematics. There are no specific trade names for the Cine-PC sequence as it has to be built from cine MRI sequences combined with the PC variants of each scanner.

**A.3 Ultrafast MRI**

The ultrafast MRI sequence, as used by Clark and colleagues (19), is a steady state gradient echo sequence that uses shortened TR and TE times in order to enhance contrast. In this sequence, a preparation pulse is added to the gradient echo sequence along with reduced TE and TR which results in ultrafast imaging. These sequences are based on FLASH (fast low-angle shot) in Siemens, T1-FFE (T1 weighted fast field echo) in Philips, and SPGR (spoiled gradient-recalled) in GE scanners. The spatial resolution of such sequences is not high and a shortened TR also reduces the contrast between tissues, reducing its use as a diagnostic tool. However, since enough contrast is produced between bone and surrounding tissues, Clarke et al (19) used it to determine ankle joint kinematics. Muhle and colleagues (20) compared cine MRI with ultrafast MRI and static MRI to evaluate patellar motion from 30° flexion to full extension (0°). For the three outcome variables studied, . patellar tilt angle, lateral patellar displacement, and bisect offset, there was no significant difference between cine MRI and ultrafast MRI data.

**A.4 Kinematic MRI**

Kinematic MRI sequence uses a fast, spoiled gradient echo pulse sequence and can acquire one image per second during active movement. This sequence is a variant of the Cine MRI sequence discussed above and typically called GRASS (gradient-recalled acquisition in the study state) in the GE scanner, FFE (fast field echo) in the Philips scanner, and FISP (fast imaging with steady-state precession) in the Siemens scanner. Using this sequence, Powers and colleagues (21) reported the repeatability of measuring patellar morphology for two trials. However, dynamic images were acquired only in single slices, making it impossible to accurately determine 3D joint kinematics. Thus, this sequence is less likely to be used for determining 3D joint kinematics.

**A.5 Velocity Encoded PC MRI**

Velocity encoding is a parameter to be specified while conducting a PC MRI study and relates to the highest velocities observed during the motion inside the scanner. The PC sequence “accumulates the phase of moving spins proportional to their velocities and to the magnitude of velocity encoding field gradients” (22). The velocity encoding parameter thus adjusts the strength of bipolar gradients so that the maximum velocity selected corresponds to a 180° phase shift in the data. The larger the gradients, the more dephasing occurs corresponding to less velocity encoding (23). Thus, the velocity of each pixel in the image can be measured. However, magnetic field heterogeneity induced by eddy currents cause errors in the quantification of such velocities (24). These are gated gradient recalled echo sequences with low TE and TR values. They are typically developed from Fast GRE sequences in GE. The trade names could not be identified for other scanners. It appears that the previously-termed velocity encoded sequence is now known as Cine-PC MRI, as the references in this review use these names for both the techniques.

**A.6 Real-time MRI and real-time PC MRI**

Real-time MRI acquires a time series of single or multiple image slices in only one motion cycle and the velocities that are measured are not averaged over multiple cycles of motion. In contrast to cine PC MRI, by only acquiring images over one motion cycle, real-time MRI is not associated with subject fatigue, allowing subjects with conditions that would prevent movement repetition to be evaluated. Fatigue is also minimized as image plane data can be acquired quickly with real-time MRI and can be reconstructed with image display rates of 24 frames/sec. In addition, the imaging plane can be continuously defined and updated in real time to continue tracking an object if motion out of the imaging plane occurs. Real-time sequence is a gradient echo based fast imaging sequence. This sequence is typically built on a balanced steady-state gradient echo pulse sequence (TrueFISP in Siemens, balanced FFE in Philips, and FIESTA in GE) or spoiled gradient echo sequence (FLASH in Siemens, T1-FFE in Philips, and SPGR in GE). Steady-state incorporates steady magnitude of longitudinal and transverse magnetization and they are ‘balanced’ by balancing the gradients in all three planes, thereby rendering them motion-insensitive (25).

The real-time PC sequence is a combination of real-time color flow MRI (26) and standard PC sequence (27), as used by Asakawa and colleagues to compare its feasibility in determining skeletal muscle velocity with the Cine-PC sequence. This technique showed accuracies up to 2cm/sec in comparison with Cine-PC MRI techniques, which has reported translational and rotational accuracies of 0.3mm and 0.46° when the joint is moved up to 30 cycles/minute. The major advantage of real-time PC over cine-PC MRI technique is that the subject only needs to perform the motion once.

**A.7 Spin tag or Tagged MRI**

Spin tag or tagging MRI tags the tissue under consideration with RF pulses, which in turn inverts spatially separated thin bands of protons and allows motion visualization as distorted tagged lines in subsequent temporal phases. Two tagging methods exist, one based on the principle of tagging a tissue of interest with RF saturation pulses to produce discrete bands of altered signal intensity as explained by Zerhouni (28), and another on spatial modulation of magnetization (SPAMM) tagged MRI with two RF pulses and gradient applied during the time between them (29). The original tagging sequences were developed using gated spin-echo pulse sequences, but later developed using gated gradient echo based sequences such as GRASS (gradient-recalled acquisition in steady state) (GE) (30), or TFE (turbo field echo) (Philips) (31). While spin-tag permits direct visualization of tissue motion, it suffers from fading of the tag lines due to T1 relaxation and requires complex post processing to derive the displacement of each pixel. An early drawback of having to produce repeated motions to achieve segmented acquisitions in SPAMM imaging was resolved by Moerman (31) by developing and validating a dynamic SPAMM method that required only three repetitions. Because of the nature of this sequence, it is only used to determine soft tissue deformations – in this case, skeletal muscle. A comparison between spin tag and phase contrast sequences showed a very good agreement between the two techniques (22).

1. Hornak JP. The Basics of MRI. https://www.cis.rit.edu/htbooks/mri/. Accessed February 17, 2017.

2. Elster AD, Burdette JH. Questions and Answers in Magnetic Resonance Imaging, 2e. 2 edition. St. Louis: Mosby; 2000.

3. Kumar A, Welti D, Ernst RR. NMR Fourier zeugmatography. 1975. J Magn Reson San Diego Calif 1997. 2011;213(2):495–509.

4. Bernstein MA, King KF, Zhou XJ. Handbook of MRI Pulse Sequences. 1 edition. Amsterdam ; Boston: Academic Press; 2004.

5. Bitar R, Leung G, Perng R, et al. MR Pulse Sequences: What Every Radiologist Wants to Know but Is Afraid to Ask. RadioGraphics. 2006;26(2):513–537.

6. Westbrook C. MRI at a Glance. 3 edition. Chichester, West Sussex ; Ames, Iowa: Wiley-Blackwell; 2016.

7. Runge VM, Nitz WR. The Physics of Clinical MR Taught Through Images. 2 edition. New York: Thieme; 2008.

8. Doty FD. MRI Gradient Coil Optimization. Spatially Resolv Magn Reson. Wiley-VCH Verlag GmbH; 1998. p. 647–674http://dx.doi.org/10.1002/9783527611843.ch60.

9. Jensen ER, Morrow DA, Felmlee JP, Odegard GM, Kaufman KR. Error analysis of cine phase contrast MRI velocity measurements used for strain calculation. J Biomech. 2015;48(1):95–103.

10. Spees WM, Buhl N, Sun P, Ackerman JJH, Neil JJ, Garbow JR. Quantification and compensation of eddy-current-induced magnetic-field gradients. J Magn Reson San Diego Calif 1997. 2011;212(1):116–123.

11. Lingamneni A, Hardy PA, Powell KA, Pelc NJ, White RD. Validation of cine phase-contrast MR imaging for motion analysis. J Magn Reson Imaging JMRI. 1995;5(3):331–338.

12. Kunz RP, Oellig F, Krummenauer F, et al. Assessment of left ventricular function by breath-hold cine MR imaging: Comparison of different steady-state free precession sequences. J Magn Reson Imaging JMRI. 2005;21(2):140–148.

13. Dorsay TA, Youngberg RA, Orr FE. Cine MRI diagnosis and posttherapeutic evaluation of an adherent TMJ disc: a case report. J Oral Maxillofac Surg Off J Am Assoc Oral Maxillofac Surg. 1994;52(11):1220–1222.

14. Langner I, Fischer S, Eisenschenk A, Langner S. Cine MRI: a new approach to the diagnosis of scapholunate dissociation. Skeletal Radiol. 2015;44(8):1103–1110.

15. Tajiri Y, Nakamura K, Matsushita T, Ohe T, Okazaki H, Nagano A. A positioning device to allow rotation for cine-MRI of the distal radioulnar joint. Clin Radiol. 1999;54(6):402–405.

16. Drace JE, Pelc NJ. Measurement of skeletal muscle motion in vivo with phase-contrast MR imaging. J Magn Reson Imaging JMRI. 1994;4(2):157–163.

17. Behnam AJ, Herzka DA, Sheehan FT. Assessing the accuracy and precision of musculoskeletal motion tracking using cine-PC MRI on a 3.0T platform. J Biomech. 2011;44(1):193–197.

18. Sheehan FT, Seisler AR, Siegel KL. In vivo talocrural and subtalar kinematics: a non-invasive 3D dynamic MRI study. Foot Ankle Int. 2007;28(3):323–335.

19. Clarke EC, Martin JH, d’Entremont AG, Pandy MG, Wilson DR, Herbert RD. A non-invasive, 3D, dynamic MRI method for measuring muscle moment arms in vivo: demonstration in the human ankle joint and Achilles tendon. Med Eng Phys. 2015;37(1):93–99.

20. Muhle C, Brossmann J, Heller M. [Functional MRI of the femoropatellar joint]. Radiol. 1995;35(2):117–124.

21. Powers CM, Shellock FG, Pfaff M. Quantification of patellar tracking using kinematic MRI. J Magn Reson Imaging JMRI. 1998;8(3):724–732.

22. Sinha S, Hodgson JA, Finni T, Lai AM, Grinstead J, Edgerton VR. Muscle kinematics during isometric contraction: development of phase contrast and spin tag techniques to study healthy and atrophied muscles. J Magn Reson Imaging JMRI. 2004;20(6):1008–1019.

23. Lotz J, Meier C, Leppert A, Galanski M. Cardiovascular flow measurement with phase-contrast MR imaging: basic facts and implementation. Radiogr Rev Publ Radiol Soc N Am Inc. 2002;22(3):651–671.

24. Drace JE, Pelc NJ. Tracking the motion of skeletal muscle with velocity-encoded MR imaging. J Magn Reson Imaging JMRI. 1994;4(6):773–778.

25. Chavhan GB, Babyn PS, Jankharia BG, Cheng H-LM, Shroff MM. Steady-state MR imaging sequences: physics, classification, and clinical applications. Radiogr Rev Publ Radiol Soc N Am Inc. 2008;28(4):1147–1160.

26. Nayak KS, Pauly JM, Kerr AB, Hu BS, Nishimura DG. Real-time color flow MRI. Magn Reson Med. 2000;43(2):251–258.

27. Pelc NJ, Herfkens RJ, Shimakawa A, Enzmann DR. Phase contrast cine magnetic resonance imaging. Magn Reson Q. 1991;7(4):229–254.

28. Zerhouni EA, Parish DM, Rogers WJ, Yang A, Shapiro EP. Human heart: tagging with MR imaging--a method for noninvasive assessment of myocardial motion. Radiology. 1988;169(1):59–63.

29. Axel L, Dougherty L. MR imaging of motion with spatial modulation of magnetization. Radiology. 1989;171(3):841–845.

30. Niitsu M, Campeau NG, Holsinger-Bampton AE, Riederer SJ, Ehman RL. Tracking motion with tagged rapid gradient-echo magnetization-prepared MR imaging. J Magn Reson Imaging JMRI. 1992;2(2):155–163.

31. Moerman KM, Sprengers AMJ, Simms CK, Lamerichs RM, Stoker J, Nederveen AJ. Validation of continuously tagged MRI for the measurement of dynamic 3D skeletal muscle tissue deformation. Med Phys. 2012;39(4):1793–1810.
